# Use of Small-Molecule Inhibitors of CILK1 and AURKA as Cilia-Promoting Drugs to Decelerate Medulloblastoma Cell Replication

**DOI:** 10.3390/biomedicines14020265

**Published:** 2026-01-24

**Authors:** Sean H. Fu, Chelsea Park, Niyathi A. Shah, Ana Limerick, Ethan W. Powers, Cassidy B. Mann, Emily M. Hyun, Ying Zhang, David L. Brautigan, Sijie Hao, Roger Abounader, Zheng Fu

**Affiliations:** 1 Department of Pharmacology, University of Virginia, Charlottesville, VA 22908, USA; gbn8rm@virginia.edu (S.H.F.); cgt3qv@virginia.edu (C.P.); kzg2kq@virginia.edu (N.A.S.); limerick@virginia.edu (A.L.); ewp6e@virginia.edu (E.W.P.); jcs3yh@virginia.edu (C.B.M.); wer6vp@virginia.edu (E.M.H.); 2 Department of Microbiology, Immunology and Cancer Biology, University of Virginia, Charlottesville, VA 22908, USA; yz5h@virginia.edu (Y.Z.); db8g@virginia.edu (D.L.B.); 3 Advanced Microscopy Facility, University of Virginia School of Medicine, Charlottesville, VA 22908, USA; pfa2xb@virginia.edu

**Keywords:** natural language processing (NLP), large language model (LLM), primary cilia, ciliogenesis, kinase, Alvocidib, Alisertib, drug, cancer

## Abstract

**Background/Objective:** The primary cilium is the sensory organelle of a cell and a dynamic membrane protrusion during the cell cycle. It originates from the centriole at G_0_/G_1_ and undergoes disassembly to release centrioles for spindle formation before a cell enters mitosis, thereby serving as a cell cycle checkpoint. Cancer cells that undergo rapid cell cycle and replication have a low ciliation rate. In this study, we aimed to identify cilia-promoting drugs that can accelerate ciliation and decelerate replication of cancer cells. **Methods:** To perform a comprehensive and efficient literature search on drugs that can promote ciliation, we developed an intelligent process that integrates either the GPT 4 Turbo, Gemini 1.5 Pro, or Claude 3.5 Haiku application programming interfaces (APIs) into a PubMed scraper that we coded, enabling the large language models (LLMs) to directly query articles for predefined user questions. We evaluated the performance of this intelligent literature search based on metrics and tested the effect of two candidate drugs on ciliation and proliferation of medulloblastoma cells. **Results:** Gemini was the best model overall, as it balanced high accuracy with solid precision and recall scores. Among the top candidate drugs identified are Alvocidib and Alisertib, small-molecule inhibitors of CILK1 and AURKA, respectively. Here, we show that both kinase inhibitors can effectively increase cilia frequency and significantly decrease the replication of medulloblastoma cells. **Conclusions:** The results demonstrated the potential of using cilia-promoting drugs, such as Alvocidib and Alisertib, to suppress cancer cell replication. Additionally, it shows the massive benefits of integrating accessible large language models to conduct sweeping, rapid, and accurate literature searches.

## 1. Introduction

Eukaryotic cells use a tiny cell membrane protrusion called the primary cilium to communicate with the environment [1,2,3]. They play a vital role in cell sensing and signaling, and defects in the structure and function of primary cilia have been associated with various diseases, collectively known as ciliopathies [4,5]. The primary cilium is a dynamic cellular organelle during the cell cycle. It is resorbed before cells enter mitosis, freeing centrioles to serve as spindle poles, and reformed after cells exit mitosis [6]. Thus, primary cilia are viewed as a structural barrier to mitosis [7,8]. Cancer cells have a low ciliation rate, which allows them to undergo rapid cell division and replication. It has been postulated that restoring primary cilia in cancer cells may decelerate cilia retraction and cancer cell replication [9,10,11]. There has been much interest in finding methods, particularly drugs and small-molecule inhibitors (SMIs), to increase the frequency and the length of primary cilia in cancer cells to slow their cell cycle and proliferation. In the field of cilia biology, many signaling molecules and regulatory pathways have been uncovered to control primary cilia formation and elongation [8,9], but their roles in the regulation of primary cilia and cell replication in cancer cells have not been fully elucidated. Therefore, a systematic literature search may reveal candidate cilia-modulating drugs, which we can further test for their impact on cancer cells.

Here, we attempted a comprehensive literature review to identify promising candidate drugs that can be further tested on the wet bench. The traditional literature review process consists of multiple steps, from beginning with searching scientific literature databases, to filtering inclusion criteria, to manual confirmation of results [12]. Manual search is time-consuming and prone to errors, since we would have to filter hundreds of thousands of loosely related articles. As such, many techniques currently exist to mitigate these issues, including Boolean searches, where logic such as “AND” and “OR” allow for more accurate results. For example, requiring “drugs AND cilia” would prevent drugs that impact other cellular organelles. Medical Subject Headings (MeSH) is another way to improve search results, using a hierarchical system of controlled vocabulary terms in PubMed. A popular tool that combines and automates these strategies is web scrapers, in which computer programs “scrape” websites (in this case, scientific literature databases) to perform a comprehensive review [13]. The user will often provide the search parameters for the program to use, including MeSH terms, as well as a certain logical flow of keywords to narrow in on exactly what they want to retrieve. For our scenario, we would want to create a scraper that could parse through PubMed, a vast and reputable database, to retrieve a smaller subgroup of relevant articles. Although this process drastically narrows down gigantic databases to relevant search results, all those articles still must be manually processed for the relevant information, a step that, especially for larger-scale searches, can take several weeks or longer, and that would greatly benefit from an AI approach. Our new method seeks to integrate artificial intelligence into the scraper, applying all the current techniques mentioned previously, while additionally allowing for retrieved articles to be automatically processed for information.

Recently, many of the popular large language models (LLMs) have released their APIs, providing the backbone of their model for integration into other applications for a very reasonable price [14]. As a result, there has been an influx of using those models for tasks far beyond the original purpose of a chatbot, ranging from educational tools [15] to programming assistants [16]. We thus proposed an AI-powered solution whereby scraped articles are automatically run through a large language model to be searched for the answers to user-predefined questions. This would enable a researcher to see exactly what they are searching for, with the relevant information automatically extracted and summarized.

LLM integration into the literature review process has previously been explored, but focuses primarily on the classification of articles based on inclusion criteria rather than the processing of those resulting articles [17,18,19,20]. Some solutions pertaining to the question-answering aspect do exist, but those models are trained to field-specific corpora of data [17]. One such study proposed a hybrid solution of traditional methods plus LLMs to better evaluate literature. They first used the BM25 model [21] to identify articles that align with query terms, and then fine-tuned various LLMs based on field-specific literature, such as COVID-19 data, and evaluated metrics [17]. However, these LLMs often have limitations in their capability and knowledge set, and can also be hard to employ for new users. Our approach instead seeks to identify an easy, accessible, and overarching pipeline that has the potential to automate multiple steps of the literature review process in a variety of areas, expanding upon previous methods and greatly increasing the efficiency of research (Table 1).

Using this LLM-integrated scraper is, in theory, a very attractive solution, but the following question remains: will the quality and accuracy of the automated results show true potential to advance research in biomedical fields? For us specifically, we wanted to test whether the drug(s) parsed via this method would induce a significant increase in ciliation rate to thereby reduce the replication rate of cancer cells.

In summary, promoting cilia growth with drugs or small-molecule inhibitors (SMIs) presents a promising approach to reducing the rate of cancer cell replication [22,23,24,25]. Finding these drugs via a manual search process is time-consuming and inefficient. Here, we attempted a solution of integrating a large language model into a PubMed web scraper, which will filter through the expansive database for relevant articles and then answer predefined user questions about the articles to retrieve important information. Furthermore, we attempted to validate the effects of two representative drugs on cancer cells. Such an intelligent and automated method, if dependably accurate, would significantly shorten the literature search process whilst ensuring high-quality results.

## 2. Methodology

### 2.1. LLM-Integrated PubMed Scraper

The primary package that we used to code the scraper was BeautifulSoup, which is very well-known for its ability and flexibility when web scraping. We imported that package into the Visual Studio code editor, where all the programming would be performed. Using a method included in the BeautifulSoup package, we fed the program the URL to PubMed, and within that, we included the specific search parameters for the retrieval of specific articles. The package also includes functions to query defined aspects of each article, such as its DOI link, abstract, title, and authors. These attributes were each saved as variables. For the large language model portion, we chose to use the GPT 4 Turbo, Gemini 1.5 Pro, and Claude 3.5 Haiku APIs, as they are the most widely used and accessible models. After creating an account on each respective website, we acquired an API key, which gave us access to all the methods associated with the large language models. We then fed the API keys into the Visual Studio file. Each time we called the large language model for a response, we invoked a method that takes two parameters: a system message and a human message. The specific wording of these terms varied by model, but the idea was constant across the models. The system message is the question or prompt defined by the user and answered by the API. The human message is the background data that we feed the model; in other words, it is the information provided to the API to allow it to accurately answer the system message. In our case, the human message would be portions of the articles we want information extracted from. Because, eventually, we wanted the answers to each of our questions to be displayed in a separate Excel cell, we created a function for each of the questions. The human message remains the article text, and the system message varies for each function to cover a different question. These answers are then assigned to individual variables. We finally use the xlsx package, a popular package to create and edit Excel files, to transfer the results for each article stored into the variables to an organized Excel file, which is ultimately returned to the user.

### 2.2. Fluorescence Imaging and Quantification of Primary Cilia and Mitotic Cells

Medulloblastoma cell line DAOY was maintained at 37 °C and 5% CO_2_ in Dulbecco’s modified Eagle’s medium (DMEM), supplemented with 10% fetal bovine serum. To image primary cilia, cells were fixed by 4% formaldehyde, rinsed in PBS, and then permeabilized by 0.2% Triton X-100 in PBS. After one hour in blocking buffer (3% goat serum, 0.2% Triton X-100 in PBS), cells were incubated with primary antibodies at 4 °C overnight, followed by rinses in PBS and one hour incubation with Alexa Fluor-conjugated secondary antibodies from Abcam (Cambridge, MA, USA). After multiple rinses, slides were mounted in antifade reagent containing DAPI (4′,6-diamidino-2-phenylindole) for imaging via a confocal Laser Scanning Microscopy 700 from ZEISS (Chester, VA, USA) at the UVA Advanced Microscopy Facility. Arl13B rabbit polyclonal antibody (17711-1-AP) and γ-tubulin mouse monoclonal antibody (66320-1-Ig) from Proteintech (Rosemont, IL, USA) were used in this study to label primary cilia and basal bodies, respectively. 

The Zen 2009 program was used with a confocal Laser Scanning Microscope 700 from ZEISS to collect z-stacks at 0.5 μm intervals to incorporate the full axoneme based on immunostaining of cilia marker Arl13b and basal body marker γ-Tubulin. All cilia were then measured in ImageJ1.54p (http://imagej.org, accessed on 21 January 2026) [26] via a standardized method based on the Pythagorean Theorem, in which cilia length was based on the equation L2 = z2 + c2, in which “c” is the longest flat length measured of the z slices, and “z” is the number of z slices in which the measured cilia were present, multiplied by the z-stack interval (0.5 μm).

Phospho-Histone H3 [pSer10] rabbit polyclonal antibody (H0412) from Sigma (Saint Louis, MI, USA) was used to label mitotic cells. To calculate the mitotic rate, we counted the number of mitotic cells (phospho-H3 positive) and divided it by the total number of cells (DAPI positive). Mitotic rates of medulloblastoma cells treated with small-molecule inhibitors were compared with the DMSO control. 

### 2.3. Drug Treatment and Crystal Violet Assay for Cell Viability

Alvocidib (Cat# S2679) and Alisertib (Cat# S1133) were purchased from Selleckchem (Houston, TX, USA) and dissolved in DMSO at 10 mM. Cancer cells were incubated with medium containing 100 nM Alvocidib and 1 µM Alisertib to ensure full inhibition of CILK1 and AURKA, respectively [27,28]. Three days after drug treatment, floating/dead cells were removed with medium, and adherent/live cells left on the plates were stained with crystal violet dye (Cat# C6158) from Millipore Sigma (Burlington, MA, USA). After extensive rinses, cells were lysed, and dyes in the lysis buffer were quantified by spectrophotometer [29].

### 2.4. Statistical Analysis

ANOVA tests were used to analyze experimental data between pairs of group means, with a *p*-value of less than 0.05 considered significant. When ANOVA tests deemed the data to be significant, post hoc Tukey HSD tests were conducted to compare group means and determine the significance of all possible experiment group pairings. This analysis was performed with alpha values of both 0.05 and 0.01. Two-tailed Student’s *t* tests were used to compare the means of two groups. For the *t* test, *p*-values less than 0.05 were considered significant.

## 3. Results

Our main objectives in this study were two-fold. From this novel LLM-integrated web scraper, we wanted to identify the best popular large language model for conducting efficient and accurate literature reviews and identify drugs that can increase the ciliation rate and decrease the proliferation rate of cancer cells. The study was conducted in a series of steps: 1. We used LLM-integrated literature review tools to perform a comprehensive sweep of existing related publications, running the scraping and processing program once with each of the models being tested; 2. we compared the results of each model to the results produced by experts in the field regarding those same articles to acquire metrics; 3. from model results and expert recommendations, we selected the top drugs that show the most promise in promoting ciliogenesis; 4. we validated the effects of such drugs on cilia length and ciliation rates in cancer cells; and 5. we evaluated the impacts of such drugs, both individually and in combination, on cancer cell replication.

### 3.1. An Intelligent Literature Search by LLM-Integrated Scraper

**Figure 1:**
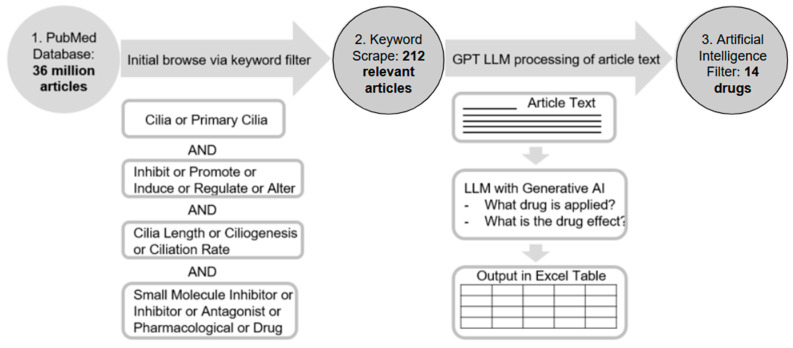
General outline of using an intelligent natural language system to search for candidate drugs modulating primary cilia. Shown are the keywords and the logic used for the retrieval of the initial group of articles and the LLM-integrated scraper process to parse through related articles.

Due to the extensive literature on scientific databases, it is challenging to execute a wide search that can encompass all the potential articles related to promoting ciliogenesis manually. Parsing through articles by hand is draining, incomprehensive, and slow, and severely limits the scope of the drugs that can be discovered. To combat such an issue, we developed a PubMed scraper that we built around the Beautiful Soup package in Python 3.14.2 and integrated the GPT 4 Turbo, Gemini 1.5 Pro, or Claude 3.5 Haiku APIs to query specific articles. The scraper operated in two distinct phases: 1. Find all relevant articles on the PubMed database using the user-provided keyword parameters; and 2. answer predefined questions using an integrated large language model (Figure 1).

The first step involved optimizing the keyword search, which was achieved by testing different combinations of related terms such as “ciliogenesis”, “drug”, or “lengthen”. The flexibility of adjusting the keywords, as well as where they show up, such as in the abstract, title, or MeSH terms, gave us multiple combinations to test, after each of which we analyzed the relatedness of the resulting articles to narrow or broaden our search as necessary. For example, if our keyword test run mainly returned articles only loosely tied to the subject (such as drugs for other organelles), we added more parameters to narrow down the results. Conversely, if only a few articles were returned in total, we knew to take away some of the requirements or adjust the logic. Ultimately, we opted for generally broader search terms, as we would much rather have unrelated articles that we can manually process after the scraper runs than have related articles that are never returned due to the specificity of terms (Figure 1). We also ensured that variations in words were considered (i.e., inhibits, inhibited, and inhibiting). For each article the scraper found, all of the basic information, such as the publication date, authors, and abstract, was saved. This initial step of extracting articles via keyword search served as a preliminary filter to create a smaller subgroup of related articles, since running the large language model on the entire PubMed database would be unfeasible and a waste of resources.

The second step was to use the integrated large language models to answer questions about each of the articles chosen by the initial keyword search. The specific questions we asked were, “What is the name of the drug?”, “What is the target of the drug?”, and “What effect(s) does the drug have on the target and primary cilia?”. For each article, the program first uses the headers to identify the abstract and results sections, as these were the only two sections we passed into the models for inquiry. Most of the information we sought was contained in these sections, so querying the entire article would give diminishing returns in terms of benefit to the added runtime ratio. So, the abstract and results sections were fed in as context for the model, as well as the three questions for the model to generate responses on. Through this method, the scraper automatically generated answers for each paper. Each article from the initial keyword-selected group, along with its basic information and answers to the questions provided by the LLM, was automatically converted to an organized Excel table format accessible immediately after the scraper finished running (Figure 1). Every row was a different article, and the columns denoted everything from the basic information about each paper to the LLM-generated answers. After optimizing the search terms, our ultimate scraper runs returned initial groups of 212 articles and took an average of five minutes to process those articles and return the Excel files. While conducting the search, we ensured that all privacy policies were adhered to. Excluded from the search were preprints and retracted articles, leaving only open-access publications accessible on PubMed.

### 3.2. Gemini 1.5 Pro Achieved the Highest Metrics and Was Identified as the Best-Performing Model

For the purpose of comparing the quality of each of the model responses, we proceeded to perform an expert-conducted manual review of the same set of articles, answering the same questions as the ones provided to the models. This process was performed independently of any model runs, and the experts did not have access to any of the model results. After completing the manual processing, we compared the expert results to each of the model results, recording true positive (*TP*), true negative (*TN*), false positive (*FP*), and false negative (*FN*) numbers accordingly. In order to be considered a true positive, the model had to have answered each of the questions correctly, including the accurate identification of the drug, the target, and the effect of the drug on cilia. A true negative occurred when both the expert and the model could not find a valid set of drug, target, and effect on cilia. A false positive occurred when the expert did not identify a valid set in the article, but the model did. A false negative occurred when the expert found a valid set in the article, but the model did not. If both the manual review and the model returned a response set, but the response from the model was incorrect, we logged both a false positive and a false negative. Using these data, we calculated accuracy, precision, recall, and F1 scores for each model. In the end, Gemini outperformed the other models in every metric (Table 2). We observed the largest differences in the recall scores, meaning that Gemini did comparatively well when it came to minimizing false negatives. The second-best model was GPT, which had roughly the same precision as Claude but a higher recall.

### 3.3. Alvocidib and Alisertib Identified as Promising Cilia-Promoting Drugs

**Figure 2:**
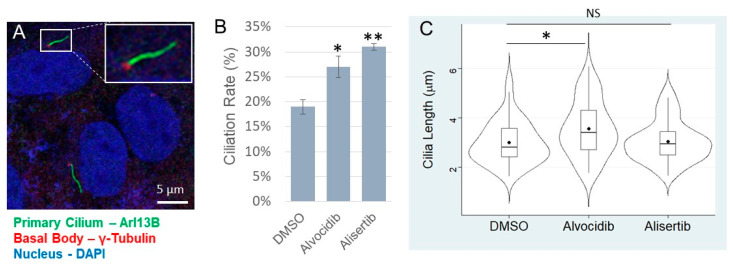
Effects of Alvocidib and Alisertib on primary cilia of medulloblastoma cells. DAOY medulloblastoma cells were treated with DMSO, Alvocidib (100 nM), or Alisertib (1 µM). (**A**) A representative confocal microscopy image showing primary cilia and basal bodies immunolabelled by Arl13B and ϒ-tubulin antibodies, respectively. DAPI stains the nucleus. Scale bar, 5 µm. (**B**) Ciliation rates were shown as mean ± SD, * *p* < 0.05, ** *p* < 0.01, and *n* = 3 independent experiments. (**C**) A Violin-Box plot showing the distribution of numerical values of cilia length (DMSO, n = 50 cilia; Alvocidib, *n* = 52 cilia; Alisertib, *n* = 58 cilia). One-way ANOVA and post hoc Tukey test were used to assess the significance of differences in group means. * Significant; NS, not significant.

The experts browsed through the answers provided by the LLM to the previously listed questions and looked specifically for drugs that targeted different pathways controlling cilia assembly or disassembly. For the drugs that showed the most promise (Table 3), we manually processed the article to ensure that all summarization information was accurate. Due to the limited time and scope for this project, we only selected two representative drugs based on their effects, well-established mechanisms of action, and the FDA-approval status (Figure 2). We chose Alvocidib, a cancer drug known to target cyclin-dependent kinases that regulate the cell cycle at high µM concentrations [30]. Such a high concentration of Alvocidib was toxic and caused strong side effects in clinical trials. However, Alvocidib instead targets a protein kinase called CILK1 (ciliogenesis-associated kinase 1) at lower nM concentrations to elongate primary cilia [27]. Recently, we have shown that the ciliary scaffold protein KATNIP (Katanin-interacting protein) can stabilize and facilitate activation of CILK1 to control ciliogenesis [31]. We propose that KATNIP-CILK1-Katanin is a new signaling axis to promote Katanin-mediated microtubule severing and cilia disassembly [31]. We also picked Alisertib, a cancer drug that targets AURKA (Aurora Kinase A) and has been shown to induce an increase in the ciliation rate [32]. AURKA phosphorylates and activates HDAC6 (histone deacetylase 6) to deacetylate tubulin and increase microtubule instability, leading to cilia disassembly [33]. Thus, inhibition of these two cilia disassembly pathways in cancer cells has the potential to impede cell proliferation by restoring or enhancing the ciliary control of the cell cycle.

### 3.4. Impact of Alvocidib and Alisertib on Primary Cilia of Medulloblastoma Cells

The papers that were associated with each drug (Alvocidib and Alisertib) showed that they statistically significantly increased ciliogenesis. However, such studies were performed on non-cancerous cells, so we needed to validate those effects on the primary cilia of cancer cells. CILK1 and AURKA are both negative regulators of ciliogenesis and can be fully inhibited by Alvocidib (100 nM) and Alisertib (1 µM), respectively. We treated DAOY medulloblastoma cells with either Alvocidib, Alisertib, or DMSO (solvent control) for 16 h and then fixed, permeabilized, and immunolabelled cells for Arl13b, the primary cilia marker, and ϒ-tubulin, the basal body marker (Figure 2A). We acquired z-stack images using a confocal immunofluorescence microscope and measured ciliation rate (Figure 2B) and cilia length (Figure 2C) using ImageJ. Compared to the DMSO control, Alvocidib induced a statistically significant increase in ciliation rate and cilia length, and Alisertib induced a statistically significant increase in cilia length (Figure 2). We conclude that both Alvocidib and Alisertib can promote the ciliation rate of medulloblastoma cells.

### 3.5. Alvocidib and Alisertib Significantly Reduce the Replication of Medulloblastoma Cells

**Figure 3:**
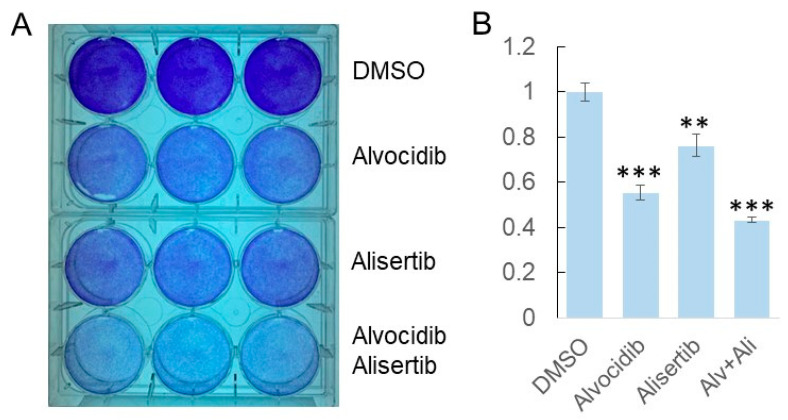
Effects of Alvocidib and Alisertib on the number of viable medulloblastoma cells. DAOY medulloblastoma cells were treated with the following drugs for 3 days in culture and assayed for cell viability: DMSO solvent control, Alvocidib (100 nM), Alisertib (1 µM), and both drugs. Viable cell counts were determined by the crystal violet dye. (**A**) A representative image of medulloblastoma cells stained by crystal violet dye in triplicate wells for each treatment; (**B**) the crystal violet dye absorbance at 590 nm relative to the DMSO control, mean ± SD, ** *p* < 0.01, *** *p* < 0.001, and *n* = 2 independent experiments.

To evaluate the effect of Alvocidib and Alisertib on medulloblastoma cells, we treated the medulloblastoma cell line DAOY with DMSO (solvent control), Alvocidib, or Alisertib. Given the possibility that CILK1 and AURKA may drive two parallel cilia disassembly pathways, we also included an additional treatment, the combination of Alvocidib and Alisertib, to test potential additive or synergistic effects of inhibiting both CILK1 and AURKA. We applied the inhibitors to fast-replicating medulloblastoma cells when the cell density reached about 50–60% confluency. After 3 days of treatment, we performed a crystal violet dye-based cell viability assay to determine the drug effects (Figure 3A). Our findings indicate that relative to DMSO control, Alvocidib induced a 40% decrease in the total cell number, Alisertib induced a 20% decrease, and their combination induced a 60% decrease (Figure 3B). These data show that Alvocidib and Alisertib significantly decrease the number of replicating medulloblastoma cells.

**Figure 4:**
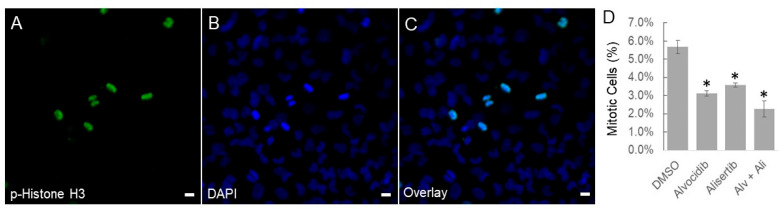
Effects of Alvocidib and Alisertib on mitosis of medulloblastoma cells. DAOY medulloblastoma cells were treated with DMSO solvent control, Alvocidib (100 nM), Alisertib (1 µM), or both drugs for 24 h before fixation and immunolabelling for the mitotic marker phospho-Histone H3. Shown in (**A**–**C**) are immunofluorescence images. Scale bar, 10 µm. Shown in (**D**) is a bar graph illustrating the percentages of mitotic cells under different treatments, mean ± SD, * *p* < 0.05, and *n* = 2 independent experiments.

To examine if the decrease in viable cell counts is due to changes in cell cycle, we fixed and permeabilized medulloblastoma cells and immuno-stained a reliable mitotic marker, phospho-Histone H3, around 24 h after drug treatment (Figure 4A–C). We observed mitotic cells, such as metaphase and anaphase, shown in Figure 4A. Quantification of the percentages of mitotic cells revealed that Alvocidib and Alisertib, either individually or in combination, caused a statistically significant decrease in mitosis of medulloblastoma cells relative to the DMSO control (Figure 4D). Dual inhibition produced a modest additive effect on the mitotic rate (Figure 4D), similar to their additive effect on the viable cell counts (Figure 3B). These data together support the hypothesis that cilia-promoting drugs decelerate medulloblastoma cell replication.

## 4. Discussion

Our study results show that the drugs discovered by this method of LLM-integrated web scraping, Alvocidib and Alisertib, can significantly promote ciliogenesis and reduce the replication of medulloblastoma cells. Under a microscope, we observed a very small number of floating dead cells after drug treatment, indicating that these drugs served primarily to hinder the cell cycle and cell replication rather than to cause massive cell death. This corroborates the mechanism of primary cilia described in the introduction as a key structural checkpoint to cell cycle and replication. Given the goal of our wet bench work in this study is to provide proof-of-principle, we focused our tests on Alvocidib and Alisertib in medulloblastoma cells because we were able to determine the effectiveness of these two drugs to promote ciliogenesis of DAOY medulloblastoma cells. Although our data are promising, more research must be carried out to determine the applicability of different cilia drug combinations to many other types of cancers, both in vitro and in vivo. For example, although Alvocidib and Alisertib can reduce replication of MCF-7 and MDA-MB-231 breast cancer cells (Figure S1), the extent of their effects in these two breast cancer cell lines varied greatly. They also failed to produce any additive effect in both breast cancer cell lines, in contrast to DAOY medulloblastoma cells (Figure 3) and A549 lung adenocarcinoma cells (Figure S1). It is worth pointing out that the assembly and disassembly of primary cilia, coupled with cell cycles, are intricate and highly regulated processes [46]. There is a significant gap in our knowledge of the molecular mechanisms by which cancer cells disable the ciliary control of cell cycles. Therefore, it would be imperative to better understand the underlying pathways and mechanisms of action of these cilia-promoting drugs in different cancer cell contexts, providing the molecular basis to expand on their applications.

One possible concern for the application of cilia-promoting drugs is their cytotoxicity on non-cancerous cells. We thus tested the effects of Alvocidib and Alisertib on non-cancerous HEK293 human embryonic kidney cells (Figure S2). The effect of Alvocidib compared with the DMSO control is not statistically significant. Alisertib and the combination of Alisertib and Alvocidib produced only a modest reduction (10–20%) in the total viable cell counts. These preliminary results suggest slight cytotoxicity of these two drugs on normal cells. To minimize the concern for off-target cytotoxic effects on normal cells, we will explore targeted delivery and controlled release of cilia-promoting drugs to cancer cells in our future in vivo studies.

Our findings also underscore the strong potential of integrating artificial intelligence-assisted scrapers into literature review and biomedical research, though further studies must be conducted to support more widespread positive LLM performance. In addition to providing accurate answers to the questions posed by the user that enabled identification of the drugs to promote ciliogenesis, the LLM-backed scraper performed those tasks at rapid speeds. The scraper parsed through the 212 articles it located in just over 5 min, meaning that in less than a second, an article had its basic information extracted, it was parsed thoroughly by the LLM to locate answers to user questions, and then that answer was generated and saved to the Excel sheet. Moreover, due to the nature of those specific answers being pre-generated when we opened the Excel sheet, it only took a couple of hours to identify those top drugs and complete the literature review process. By contrast, a manual review process would require one to click into each individual article and then parse the article for relevant information. Oftentimes, one must go beyond the abstract to find some of the finer details necessary for the search. Compounded with breaks and inconsistencies, this process could easily take days or even weeks. Our proposed method drastically reduces this time with minimal sacrifices to quality.

Additionally, our approach is very versatile in terms of applying to various other areas of study. This is due to our overarching framework’s flexibility of using a keyword search-centered web scraper to select a subset of research and then ask questions to synthesize article information. Within this process, there are two major places where adjustments can be made to alter the content of the literature search. The first is the set of keywords and logic used to conduct the initial filtration of articles, determining which field the literature search is being performed in. Furthermore, there is much flexibility in this step regarding the specificity of the keywords chosen. Fewer keywords and logic will lend themselves to a broader search that encompasses more articles, while more keywords with stricter logic will return fewer and more specific papers. The second place in the process is the selection of questions given to the LLM for the analysis of the keyword-selected articles. Questions can range from summarizing the paper to finding a very specific attribute of the study that the reviewer is looking for.

Despite the significant enhancement in speed, the LLM-integrated literature search aspect of our study also has certain limitations. As stated previously, the manual selection of keywords and questions dictates the results that are returned. In our study, professionals in the field validated the keywords used for the initial search and the questions that were used by the LLM for the detailed processing step. Even then, we had to perform several searches and compare the results to judge the best combination of keywords and questions. This manual trial-and-error process for the literature search means that there is no guarantee that the specific keywords and questions—as well as the number of keywords and questions we used—are optimized. This lack of objectivity and heavy reliance on human input introduces bias into the process, enabling the possible omission of relevant studies. One alternative is to use generative artificial intelligence methods to create optimal Boolean searches, but the current state of those methods is very imperfect. A recent study conducted by Wang et al. [47] prompted ChatGPT to generate Boolean searches based on a field of interest. Though certain results were promising, the team also experienced many instances of nonexistent MeSH terms, as well as inconsistencies between different executions of the same prompt. Adam et al. [48] sought to expand upon these results by training models with large amounts of relevant data, but they concluded that the results were still too inconsistent to implement without manual verification. Conversely, an older but generally more consistent method involves some form of word or phrase tokenization and subsequently training a neural network on that data [49]. Such an example includes a model that takes a word and quantifies whether or not that word would be beneficial to expanding a query. This could be especially helpful for optimizing query length. Thus, in future studies, we hope to automate this segment of the process with the aforementioned artificial intelligence or machine learning-based solutions that operate consistently, giving us the maximum amount of confidence that the results and answers produced are the best suited for that specific literature search scenario. An example of such an implementation could include automatically assigning resulting articles a score based on word and phrase relevancy, and having a model optimize that score by experimenting with various keyword combinations.

Another aspect that shows much room for improvement is the answers generated by LLMs when processing the articles. The overall solid metrics show much potential for these models, and it must be noted that these scores are considerably more difficult to achieve than a simple classification task, because the responses to all three questions must be accurate to count. Many of the false positive cases by the model were due to mislabeling non-drugs as drugs, since, in context, they may have seemed to take on the role of a drug. In other cases, the models falsely identified drug–target combinations that were invalid because the drug did not directly affect the target. Most of the false negative cases were due to the limited scope of the models, and especially due to a decreasing accuracy in the middle of long texts. As such, there is still room for improvement in terms of identifying this information when it is contained deep within a text’s body. Other minor errors included the accidental switching of drug and target when converting to the Excel sheet or failing to directly relate the consequences of the drug–target combination on cilia. Thus, a human professional is still crucial to ensuring the validity of articles and drugs.

## 5. Conclusions

Our study demonstrated how to use an intelligent language system to conduct a fast and comprehensive literature search for effective cilia-promoting drugs. We validated that two such drugs, small-molecule inhibitors for CILK1 and AURKA, achieved the desired goal of increasing ciliation and reducing replication of medulloblastoma cells. Our findings implicated a strong potential of cilia-promoting drugs in cancer therapy. A major challenge to our future investigations is to determine the optimal drug combinations for restoring or enhancing ciliation in each specific type of cancer and to minimize off-target cytotoxicity on normal cells. Such findings also highlight the incredible potential of these newly emerging and widely accessible large language models to conduct fast and accurate literature searches. However, further research must be conducted to confirm applications in other fields, improve upon the current shortcomings of LLMs, and find objective procedures to automate the optimization of keywords. Despite these areas for further improvement, this method of LLM-integrated literature scrapers presents significant potential for use as an assistive tool for biomedical research.

## Figures and Tables

**Table 1:** Outline of the traditional literature review process, with steps to be automated by the proposed pipeline in bold.

The Literature Review Workflow
1. Formulating research questions and objectives
**2. Searching scientific databases for relevant literature**
**3. Screening for inclusion**
4. Assessing the quality of scraped articles
**5. Extracting data**
6. Analyzing and synthesizing data

**Table 2:** The comparison between three LLMs in an intelligent literature review.

	GPT 4 Turbo	Gemini 1.5 Pro	Claude 3.5 Haiku
**Accuracy** $\left(\frac{TP+TN}{TP+TN+FP+FN}\right)$	0.86	0.88	0.85
**Precision** $\left(\frac{TP}{TP+FP}\right)$	0.80	0.85	0.80
**Recall** $\left(\frac{TP}{TP+FN}\right)$	0.73	0.77	0.67
**F1** $\;\left(2*\frac{Precision*Recall}{Precision+Recall}\right)$	0.76	0.81	0.73

**Table 3:** Summary of top drugs and their targets and effects on primary cilia, and their FDA status.

Drug [Ref]	Target	Effect on Primary Cilia	FDA and Clinical Trial Status
SR9011 [34]	REV-ERB	Cilia length ↑	N/A
Cytochalasin D [35]	Actin	Cilia length and frequency ↑	N/A
Rotenone [36]	Mitochondrial complex 1	Cell type-dependent effect on cilia length and frequency	N/A
Alvocidib [27]	CILK1	Cilia length ↑	Clinical trials for various types of cancer; orphan drug approval for leukemia
Fenoldopam [37]	Dopamine-like 1	Cilia length ↑	Clinical trials for acute renal failure and hypertension; FDA approved for hypertension in 1997, currently discontinued
Etoposide [38]	Topoisomerase II	Cilia frequency ↑	FDA approved for small-cell lung cancer and testicular cancer
AK-1 [39]	SIRT2	Cilia frequency ↑	N/A
1-methyl-4-phenylpyridinium [40]	Mitochondrial complex 1	Cilia length and frequency ↑	N/A
Y27632 [41]	Rho kinase	Cilia length ↑	N/A
Alisertib [28]	AURKA	Cilia frequency ↑	Clinical trials for various types of cancer; granted FDA orphan drug designation for small-cell lung cancer
Tubacin [42]	HDAC6	Cilia length and frequency ↑	N/A
Jasplakinolide [43]	Actin	Cilia frequency ↑	N/A
Brefeldin A [44]	CK1ɛ-Dvl2 complex	Cilia length ↑	N/A
Ent-Kaurane diterpenoids [45]	Smo	Cilia length ↑	N/A

Note: REV-ERB (NR1D1): Nuclear receptor subfamily 1 group D member 1; CILK1: Ciliogenesis-associated kinase 1; SIRT2: Sirtuin 2; AURKA: Aurora kinase A; HDAC6: Histone deacetylase 6; CK1ɛ: Casein kinase I isoform epsilon; Dvl2: disheveled segment polarity protein 2; and Smo: Smoothened. Drug status was acquired from Drugs@FDA and ClinicalTrials.gov. ↑ denotes an increase.

## Data Availability

The original contributions presented in the study are included in the article/Supplementary Materials, and further inquiries can be directed to the corresponding authors.

## Supplementary Materials

The following supporting information can be downloaded at https://www.mdpi.com/article/10.3390/biomedicines14020265/s1: Figure S1: Effects of Alvocidib and Alisertib on breast and lung cancer cell viability. Figure S2: Effects of Alvocidib and Alisertib on normal human embryonic kidney cells. Figures S3/S4/S5: Negative controls and original microscopy images. The scraper code with LLM is available at https://github.com/uvapharm/cilia_scraper, deposited on 30 September 2025.

## References

[1] Fry A.M., Leaper M.J., Bayliss R. (2014). The primary cilium. Organogenesis.

[2] Singla V., Reiter J.F. (2006). The primary cilium as the cell’s antenna: Signaling at a sensory organelle. Science.

[3] Malicki J.J., Johnson C.A. (2017). The Cilium: Cellular Antenna and Central Processing Unit. Trends Cell Biol..

[4] Reiter J.F., Leroux M.R. (2017). Genes and molecular pathways underpinning ciliopathies. Nat. Rev. Mol. Cell Biol..

[5] Badano J.L., Mitsuma N., Beales P.L., Katsanis N. (2006). The ciliopathies: An emerging class of human genetic disorders. Annu. Rev. Genom. Hum. Genet..

[6] Plotnikova O.V., Pugacheva E.N., Golemis E.A. (2009). Primary Cilia and the Cell Cycle. Methods Cell Biol..

[7] Goto H., Inoko A., Inagaki M. (2013). Cell cycle progression by the repression of primary cilia formation in proliferating cells. Cell. Mol. Life Sci..

[8] Izawa I., Goto H., Kasahara K., Inagaki M. (2015). Current topics of functional links between primary cilia and cell cycle. Cilia.

[9] Goto H., Inaba H., Inagaki M. (2017). Mechanisms of ciliogenesis suppression in dividing cells. Cell. Mol. Life Sci..

[10] Halder P., Khatun S., Majumder S. (2020). Freeing the brake: Proliferation needs primary cilium to disassemble. J. Biosci..

[11] Peixoto E., Richard S., Pant K., Biswas A., Gradilone S.A. (2020). The primary cilium: Its role as a tumor suppressor organelle. Biochem. Pharmacol..

[12] Paré G., Kitsiou S. (2017). Chapter 9 Methods for Literature Reviews. Handbook of eHealth Evaluation: An Evidence-Based Approach [Internet].

[13] Lotfi C., Srinivasan S., Ertz M., Latrous I. Web Scraping Techniques and Applications: A Literature Review. Proceedings of the SCRS Conference Proceedings on Intelligent Systems.

[14] Kublik S., Saboo S. (2023). GPT-3: The Ultimate Guide To Building NLP Products with OpenAI API.

[15] Lu C.-Y., Chen I., Cheng Y.-P., Pedaste M., Bardone E., Huang Y.-M. (2024). Leveraging OpenAI API for Developing a Monopoly Game-Inspired Educational Tool Fostering Collaborative Learning and Self-efficacy. Innovative Technologies and Learning.

[16] Finnie-Ansley J., Denny P., Becker B.A., Luxton-Reilly A., Prather J. (2022). The Robots Are Coming: Exploring the Implications of OpenAI Codex on Introductory Programming. In title of ACE ’22. Proceedings of the 24th Australasian Computing Education Conference.

[17] Yang H., Li S., Gonçalves T. (2024). Enhancing Biomedical Question Answering with Large Language Models. Information.

[18] Nassiri K., Akhloufi M.A. (2024). Recent Advances in Large Language Models for Healthcare. BioMedInformatics.

[19] Ng S.H.-X., Teow K.L., Ang G.Y., Tan W.S., Hum A. (2023). Semi-automating abstract screening with a natural language model pretrained on biomedical literature. Syst. Rev..

[20] Panagides R.K., Fu S.H., Jung S.H., Singh A., Muttikkal R.T.E., Broad R.M., Meakem T.D., Hamilton R.A. (2024). Enhancing Literature Review Efficiency: A Case Study on Using Fine-Tuned BERT for Classifying Focused Ultrasound-Related Articles. AI.

[21] Robertson S., Zaragoza H. (2009). The Probabilistic Relevance Framework: BM25 and Beyond. Found. Trends Inf. Retr..

[22] Gradilone S.A., Radtke B.N., Bogert P.S., Huang B.Q., Gajdos G.B., LaRusso N.F. (2013). HDAC6 inhibition restores ciliary expression and decreases tumor growth. Cancer Res..

[23] Qie Y., Wang L., Du E., Chen S., Lu C., Ding N., Yang K., Xu Y. (2020). TACC3 promotes prostate cancer cell proliferation and restrains primary cilium formation. Exp. Cell Res..

[24] Zingg D., Debbache J., Peña-Hernández R., Antunes A.T., Schaefer S.M., Cheng P.F., Zimmerli D., Haeusel J., Calçada R.R., Tuncer E. (2018). EZH2-Mediated Primary Cilium Deconstruction Drives Metastatic Melanoma Formation. Cancer Cell.

[25] Xiang W., Guo F., Cheng W., Zhang J., Huang J., Wang R., Ma Z., Xu K. (2017). HDAC6 inhibition suppresses chondrosarcoma by restoring the expression of primary cilia. Oncol. Rep..

[26] Schroeder A.B., Dobson E.T.A., Rueden C.T., Tomancak P., Jug F., Eliceiri K.W. (2021). The ImageJ ecosystem: Open-source software for image visualization, processing, and analysis. Protein Sci..

[27] Wang E.X., Turner J.S., Brautigan D.L., Fu Z. (2022). Modulation of Primary Cilia by Alvocidib Inhibition of CILK1. Int. J. Mol. Sci..

[28] Jeffries E.P., Di Filippo M., Galbiati F. (2019). Failure to reabsorb the primary cilium induces cellular senescence. FASEB J..

[29] Feoktistova M., Geserick P., Leverkus M. (2016). Crystal Violet Assay for Determining Viability of Cultured Cells. Cold Spring Harb. Protoc..

[30] Am S. (1999). Flavopiridol: The first cyclin-dependent kinase inhibitor in human clinical trials. Investig. New Drugs.

[31] Turner J.S., McCabe E.A., Kuang K.W., Gailey C.D., Brautigan D.L., Limerick A., Wang E.X., Fu Z. (2023). The Scaffold Protein KATNIP Enhances CILK1 Control of Primary Cilia. Mol. Cell. Biol..

[32] Malumbres M., de Castro I.P. (2014). Aurora kinase A inhibitors: Promising agents in antitumoral therapy. Expert Opin. Ther. Targets.

[33] Pugacheva E.N., Jablonski S.A., Hartman T.R., Henske E.P., Golemis E.A. (2007). HEF1-dependent Aurora A activation induces disassembly of the primary cilium. Cell.

[34] Nakazato R., Matsuda Y., Ijaz F., Ikegami K. (2023). Circadian Oscillation in Primary CILIUM Length by Clock Genes Regulates Fibroblast Cell Migration. EMBO Rep..

[35] Liu J., Leng F.-F., Gao Y.-H., He W.-F., Wang J.-F., Xian C.J., Ma H.-P., Chen K.-M. (2022). Protection of primary cilia is an effective countermeasure against the impairment of osteoblast function induced by simulated microgravity. J. Cell. Mol. Med..

[36] Moruzzi N., Valladolid-Acebes I., Kannabiran S.A., Bulgaro S., Burtscher I., Leibiger B., Leibiger I.B., Berggren P.-O., Brismar K. (2022). Mitochondrial impairment and intracellular reactive oxygen species alter primary cilia morphology. Life Sci. Alliance.

[37] Spasic M., Duffy M.P., Jacobs C.R. (2022). Fenoldopam sensitizes primary cilia-mediated mechanosensing to promote osteogenic intercellular signaling and whole bone adaptation. J. Bone Miner. Res. Off. J. Am. Soc. Bone Miner. Res..

[38] Teng Y.-N., Chang H.-C., Chao Y.-Y., Cheng H.-L., Lien W.-C., Wang C.-Y. (2021). Etoposide Triggers Cellular Senescence by Inducing Multiple Centrosomes and Primary Cilia in Adrenocortical Tumor Cells. Cells.

[39] Lim J., Son J., Ryu J., Kim J.-E. (2020). SIRT2 Affects Primary Cilia Formation by Regulating mTOR Signaling in Retinal Pigmented Epithelial Cells. Int. J. Mol. Sci..

[40] Bae J.-E., Kang G.M., Min S.H., Jo D.S., Jung Y.-K., Kim K., Kim M.-S., Cho D.-H. (2019). Primary cilia mediate mitochondrial stress responses to promote dopamine neuron survival in a Parkinson’s disease model. Cell Death Dis..

[41] Kakiuchi A., Kohno T., Kakuki T., Kaneko Y., Konno T., Hosaka Y., Hata T., Kikuchi S., Ninomiya T., Himi T. (2019). Rho-kinase and PKCα Inhibition Induces Primary Cilia Elongation and Alters the Behavior of Undifferentiated and Differentiated Temperature-sensitive Mouse Cochlear Cells. J. Histochem. Cytochem..

[42] Rowson D.T., Shelton J.C., Screen H.R.C., Knight M.M. (2018). Mechanical loading induces primary cilia disassembly in tendon cells via TGFβ and HDAC6. Sci. Rep..

[43] Nagai T., Mizuno K. (2017). Jasplakinolide induces primary cilium formation through cell rounding and YAP inactivation. PLoS ONE.

[44] Lee U., Kim S.-O., Hwang J.-A., Jang J.-H., Son S., Ryoo I.-J., Ahn J.S., Kim B.Y., Lee K.H. (2017). The Fungal Metabolite Brefeldin A Inhibits Dvl2-Plk1-Dependent Primary Cilium Disassembly. Mol. Cells.

[45] Jiang S., Du J., Kong Q., Li C., Li Y., Sun H., Pu J., Mao B. (2015). A Group of ent-Kaurane Diterpenoids Inhibit Hedgehog Signaling and Induce Cilia Elongation. PLoS ONE.

[46] Wang L., Dynlacht B.D. (2018). The regulation of cilium assembly and disassembly in development and disease. Development.

[47] Wang S., Scells H., Koopman B., Zuccon G. (2023). Can ChatGPT Write a Good Boolean Query for Systematic Review Literature Search?. arXiv.

[48] Adam G.P., DeYoung J., Paul A., Saldanha I.J., Balk E.M., Trikalinos T.A., Wallace B.C. (2024). Literature search sandbox: A large language model that generates search queries for systematic reviews. JAMIA Open.

[49] Imani A., Vakili A., Montazer A., Shakery A. (2018). Deep Neural Networks for Query Expansion using Word Embeddings. arXiv.

