# Supplementary material for: Use of Small-Molecule Inhibitors of CILK1 and AURKA as Cilia-Promoting Drugs to Decelerate Medulloblastoma Cell Replication
Source: Biomedicines. 2026 Jan 24;14(2):265. doi: 10.3390/biomedicines14020265 (PMC12938322; doi:10.3390/biomedicines14020265)

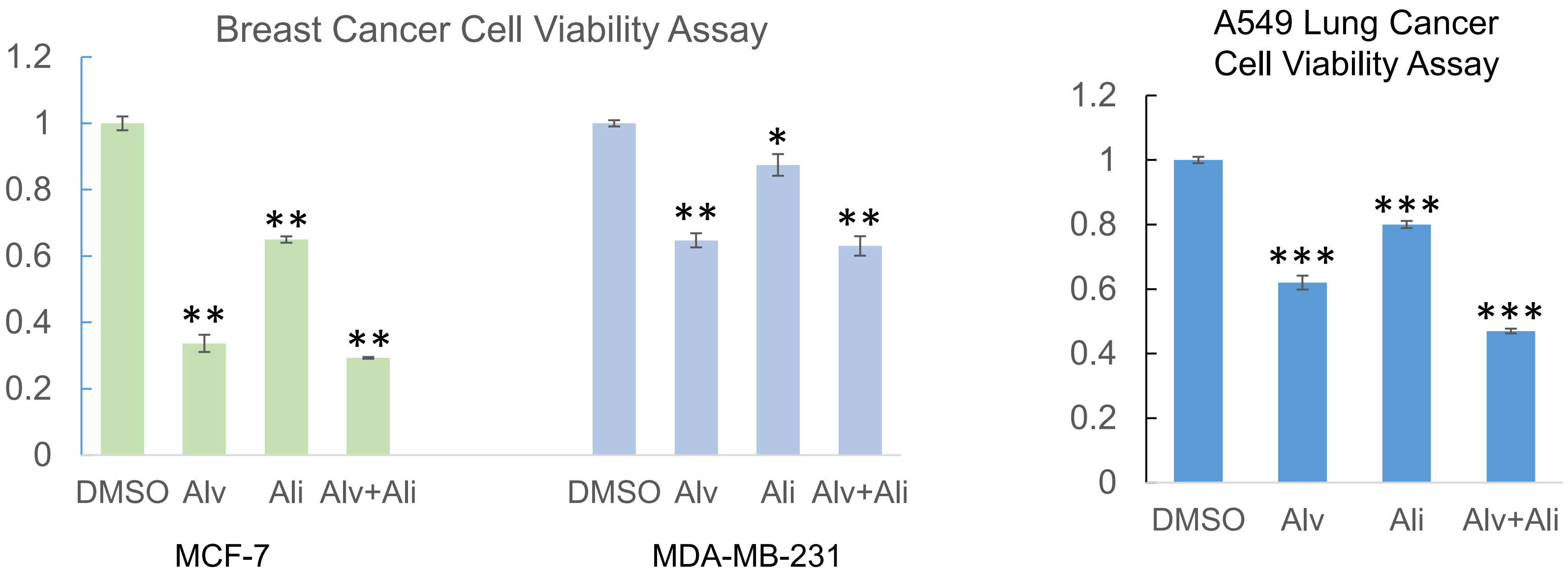


**Figure S1**: Effects of Alvocidib and Alisertib on breast and lung cancer cell viability. We treated MCF-7 and MDA-MB-231 breast cancer cells or A549 lung adenocarcinoma cells with DMSO (control) or Alvocidib (Alv, 100 nM) or Alisertib (Ali, 1 μM) or both for 72 hours and removed dead/detached cells and stained the remaining adherent cells with crystal violet. Shown is the crystal violet dye absorbance at 590 nm relative to the DMSO control, mean ± SD, n = 3 wells, *P<0.05, **P<0.01, ***P<0.001.

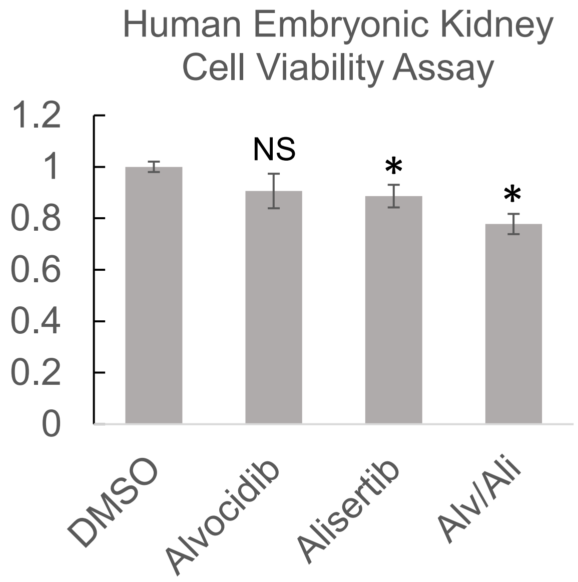


**Figure S2**: Effects of Alvocidib and Alisertib on HEK293 human embryonic kidney cells. We treated HEK293 cells with DMSO (control) or Alvocidib (Alv, 100 nM) or Alisertib (Ali, 1 μM) or both for 72 hours and removed dead/detached cells and stained the remaining adherent cells with crystal violet. Shown is the crystal violet dye absorbance at 590 nm relative to the DMSO control, mean ± SD, n = 3 wells, *P<0.05, **P<0.01, ***P<0.001, NS = not significant.

**Figure S3**: Negative Controls for Figure 2A - without Primary Antibodies

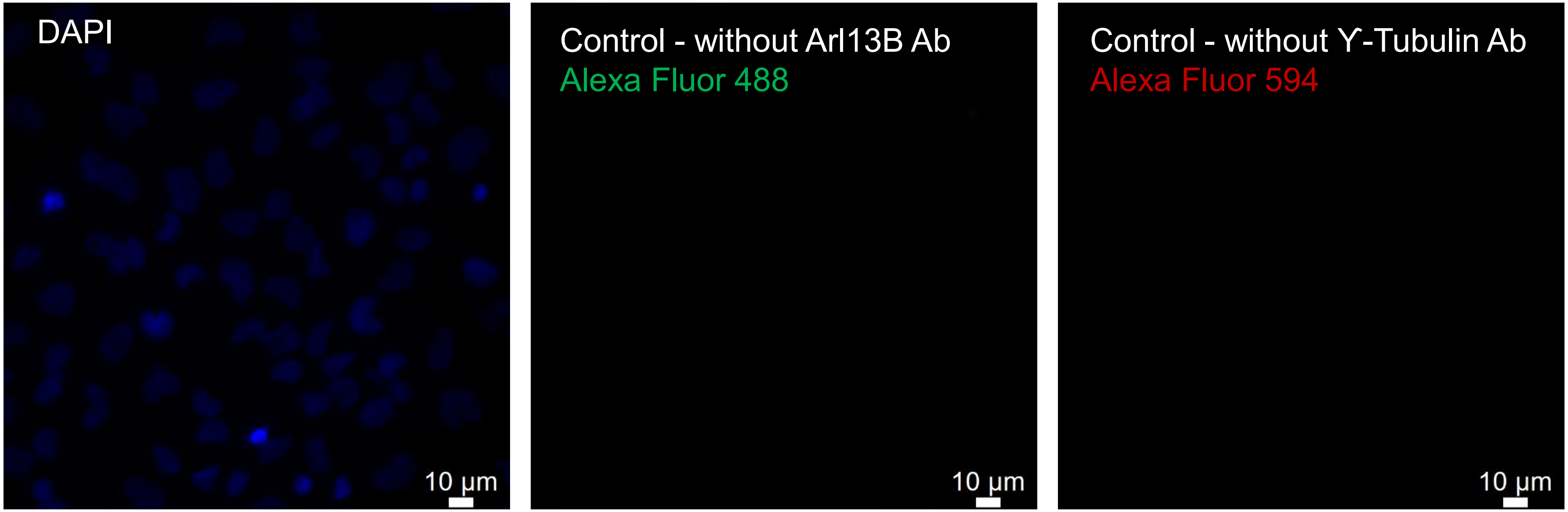

**Figure S4**: Original Microscopy Image for Figure 2A

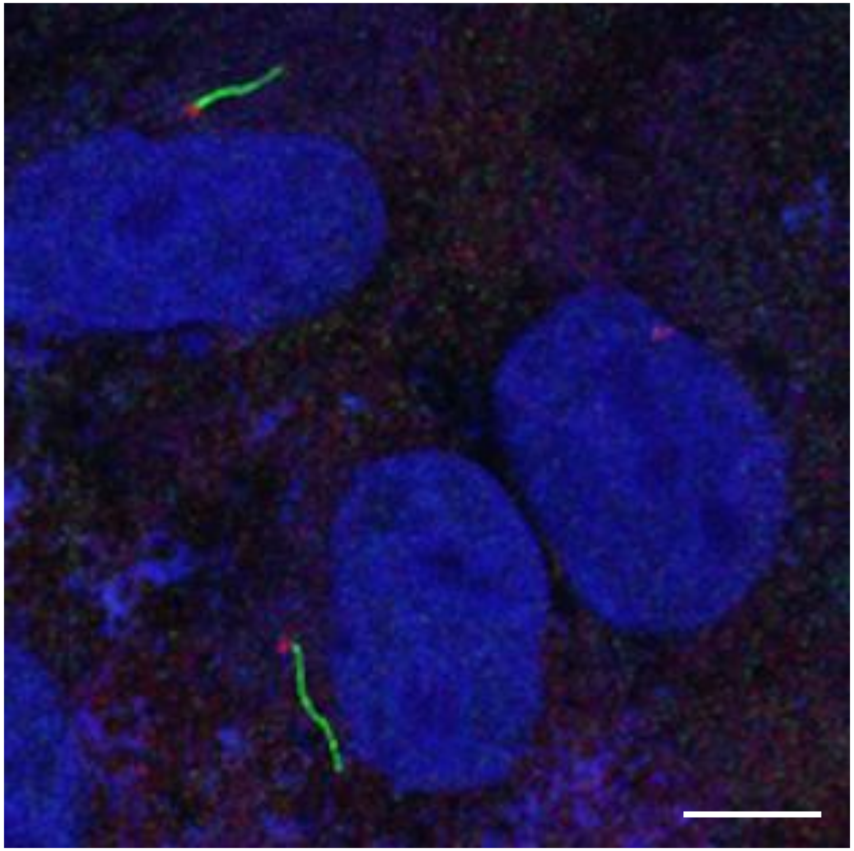

**Figure S5**: Original Microscopy Images for Figure 4

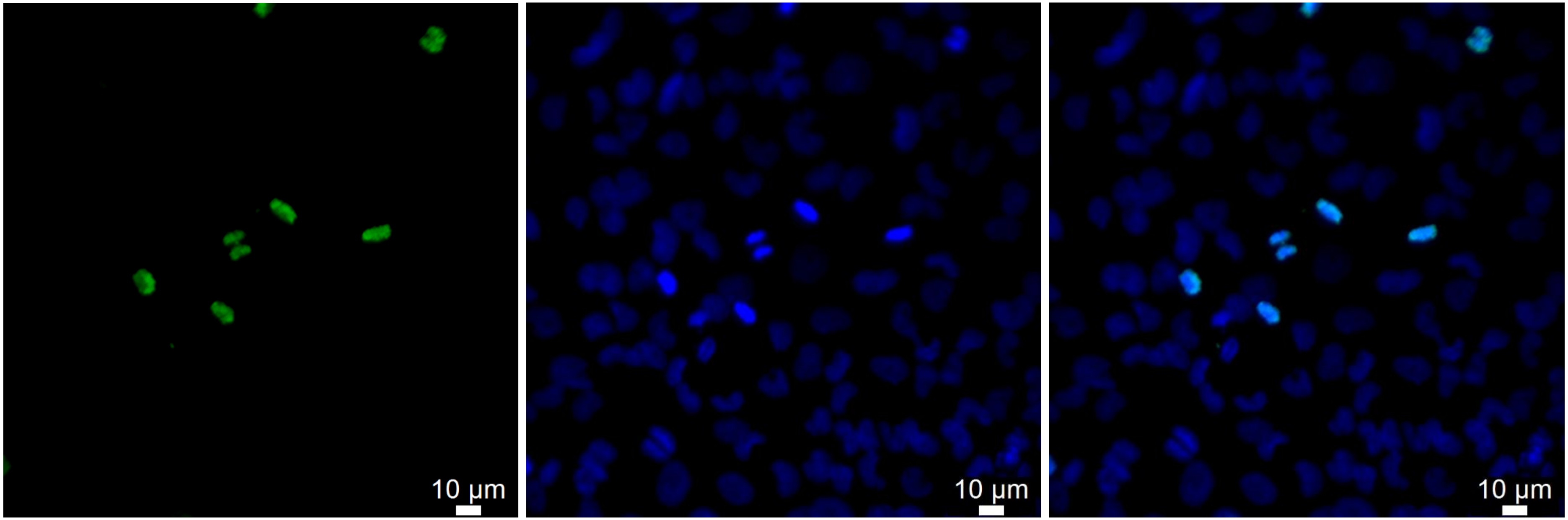

Supplement: Supplementary file 1 [file biomedicines-14-00265-s001.zip › biomedicines-3934661-supplementary.pdf]
